# Cost of introducing and delivering malaria vaccine (RTS, S/AS01_E_) in areas of seasonal malaria transmission, Mali and Burkina Faso

**DOI:** 10.1136/bmjgh-2022-011316

**Published:** 2023-04-17

**Authors:** Halimatou Diawara, Fadima Yaya Bocoum, Alassane Dicko, Ann Levin, Cynthia Lee, Fatoumata Koita, Jean Bosco Ouédraogo, Rosemonde Guissou, Seydou Yabré, Seydou Traoré, Winthrop Morgan, Clint Pecenka, Ranju Baral

**Affiliations:** 1 Malaria Research and Training Centre, University of Science Techniques and Technologies of Bamako, Bamako, Mali; 2 Chercheur en sciences sociales, Institut de Recherche en Sciences de la Santé, Ouagadougou, Burkina Faso; 3 Levin & Morgan LLC, Bethesda, Maryland, USA; 4 PATH, Seattle, Washington DC, USA; 5 Institut de Recherche en Sciences do la Sante, Bobo-Dioulasso, Burkina Faso; 6 Université Thomas Sankara, Saaba, Burkina Faso

**Keywords:** malaria, vaccines

## Abstract

**Background:**

The WHO recommends use of the RTS, S/AS01_E_ (RTS, S) malaria vaccine for young children living in areas of moderate to high *Plasmodium falciparum* malaria transmission and suggests countries consider seasonal vaccination in areas with highly seasonal malaria. Seasonal vaccination is uncommon and may require adaptations with potential cost consequences. This study prospectively estimates cost of seasonal malaria vaccine delivery in Mali and Burkina Faso.

**Methods:**

Three scenarios for seasonal vaccine delivery are costed (1) mass campaign only, (2) routine Expanded Programme on Immunisation (EPI) and (3) mixed delivery (mass campaign and routine EPI)), from the government’s perspective. Resource use data are informed by previous new vaccine introductions, supplemented with primary data from a sample of health facilities and administrative units.

**Findings:**

At an assumed vaccine price of US $5 per dose, the economic cost per dose administered ranges between $7.73 and $8.68 (mass campaign), $7.04 and $7.38 (routine EPI) and $7.26 and $7.93 (mixed delivery). Excluding commodities, the cost ranges between $1.17 and $2.12 (mass campaign), $0.48 and $0.82 (routine EPI) and $0.70 and $1.37 (mixed delivery). The financial non-commodity cost per dose administered ranges between $0.99 and $1.99 (mass campaign), $0.39 and $0.76 (routine EPI) and $0.58 and $1.28 (mixed delivery). Excluding commodity costs, service delivery is the main cost driver under the mass campaign scenario, accounting for 36% to 55% of the financial cost. Service delivery accounts for 2%–8% and 12%–23% of the total financial cost under routine EPI and mixed delivery scenarios, respectively.

**Conclusion:**

Vaccine delivery using the mass campaign approach is most costly followed by mixed delivery and routine EPI delivery approaches, in both countries. Our cost estimates provide useful insights for decisions regarding delivery approaches, as countries plan the malaria vaccine rollout.

**WHAT IS ALREADY KNOWN ON THIS TOPIC**

- Malaria parasite transmission is highly seasonal across the African Sahel subregion. WHO recommends provision of the RTS, S/AS01_E_ malaria vaccine, in areas with highly seasonal malaria or areas with perennial malaria transmission with seasonal peaks.
- Seasonal vaccine delivery may require new approaches and adaptations to existing routine childhood vaccination strategies, with potential cost consequences. No known evidence is available on the costs of seasonal delivery approaches for malaria vaccines.

**WHAT THIS STUDY ADDS**

- This is one of the first studies to examine the costs of seasonal RTS, S vaccine delivery under alternative scenarios of vaccine delivery.
- The non-vaccine economic cost of delivery per dose ranges between $0.48 and $2.12 across the three alternative scenarios considered. Vaccine delivery using a targeted mass campaign approach is most costly, followed by mixed delivery and routine EPI approaches requiring relatively fewer adaptations of existing routine immunisation programmes.

**HOW THIS STUDY MIGHT AFFECT RESEARCH, PRACTICE OR POLICY**

- Countries planning to adopt and roll out the malaria vaccine in seasonal transmission settings should consider alternative vaccine delivery approaches, as guided by the local epidemiology, for achieving the desired impact. The options have varying cost implications to governments as evidenced by the findings in this study.
- Final vaccine product price and the choice of delivery strategies will be important to assess the affordability and sustainability of delivery of the malaria vaccine.

## Introduction

Malaria parasite transmission is highly seasonal across the African Sahel subregion with more than 60% of the burden occurring during the rainy season.^1^ Clinical trials in Burkina Faso and Mali evaluating RTS, S/AS01_E_ (RTS, S) malaria vaccine use in areas with seasonal malaria transmission provided encouraging results suggesting substantial reductions in malaria disease burden by using the RTS, S vaccine alone or in combination with seasonal malaria chemoprevention (SMC).^2^ Drawing on this body of evidence, the WHO recommendation on widespread use of the RTS, S malaria vaccine invites countries to consider providing the vaccine seasonally, in areas with highly seasonal malaria or areas with perennial malaria transmission with seasonal peaks.^3 4^

RTS, S use in the seasonal context may require new approaches to vaccine delivery. Feasible and sustainable modalities of seasonal vaccine delivery are yet to be determined and will likely be country specific, necessitating adaptations to routine childhood vaccination strategies with potential cost consequences. Country decisions around adopting new interventions are guided by feasibility of implementation as well as economic and other considerations, especially in resource-constrained settings. Understanding economic factors and cost of implementation is critical to inform the value of each delivery approach and assess the modalities to help inform decision-making and planning around further use of the vaccine. Some evidence of the economic feasibility of delivering a malaria vaccine is available.^5–7^ However, there is no known evidence on the costs of seasonal delivery approaches for malaria vaccines.

This study examines the cost of introducing and delivering the malaria vaccine in Mali and Burkina Faso, countries with seasonal malaria transmission. We evaluate the costs to the health systems in these countries, under alternative delivery approaches or scenarios within the context of seasonal transmission. Findings from this analysis will allow in-country and global policymakers to better understand the cost implications of implementing a new vaccine under alternative delivery scenarios and to assess the financial and economic feasibility of each option. Additionally, the cost estimates generated from the analysis could be used to inform cost-effectiveness and budget-impact analyses of the malaria vaccine to support country decision-making on the expanded use of the vaccine in seasonal settings.

## Methods

### Study setting

Childhood vaccinations, in Burkina Faso and Mali, are managed by the respective National immunisation programmes and are delivered primarily as a routine service across all health facilities. Under routine immunisation, some outreach programmes are also regularly organised by health facilities to vaccinate eligible children closer to their communities. Additional ad hoc vaccination campaigns are organised on a need basis, for example, for polio, measles, COVID-19, and their delivery is often supported by partner agencies. Coverage of routine childhood immunisation is relatively high in both countries.

### Scope and perspective

RTS, S is not currently used as a routine intervention in Mali or Burkina Faso. We, therefore, take a prospective (cost projection) approach to project costs of malaria vaccine introduction and delivery to the health system from the perspective of the two governments. The analysis considers costs incremental to the existing immunisation programme that may be incurred under an alternative implementation scenario. Cost estimates are generated for a period of 5 years starting in 2022 and assume national introduction in both countries. Any direct expenses identified as necessary for vaccine introduction and delivery are considered financial costs to the government. Such costs may be supported by external donor agencies or through new vaccine introduction grants from Gavi, the Vaccine Alliance (Gavi) in the future.

### Target age group and schedule

The malaria vaccine is targeted for children between the ages 0–5 years. Children in their first year are considered eligible for the first dose, with the first three doses given to children at approximately monthly intervals. The current WHO recommendation is to give a four-dose schedule of the RTS, S vaccine to children approximately 12–18 months following the third dose, with an optional five-dose schedule for areas with highly seasonal malaria parasite transmission.^3^ This study followed the ongoing clinical trials in Burkina Faso and Mali^2^ looking at the benefits of the malaria vaccine given in conjunction with SMC, where over the course of 5 years, a child could receive a total of seven doses of the vaccine. While the current recommendation is to provide up to five doses of vaccine, evaluating costs of additional annual doses can provide insights into possible costs for the addition of annual doses through age 5.

### Scenarios for costing

Routine childhood immunisation programmes primarily target children under 2 years of age and deliver vaccines on an age-based schedule. Using a seasonal approach to RTS, S vaccine delivery, children would likely receive a vaccination annually up to 5 years of age, well beyond the target age group of the Expanded Programme on Immunisation (EPI). Additionally, seasonally targeted delivery at the start of the disease transmission season is desired for the vaccine to be most impactful. These elements require adaptation of current routine immunisation programmes. Alternative vaccine delivery scenarios were generated with inputs from global experts as well as extensive consultations with in-country stakeholders, including decision-makers for new vaccine introduction and malaria programme managers. The discussions also built on and drew on insights from a qualitative study on feasibility and acceptability of the RTS, S vaccine and SMC delivery in country.^8^

#### Scenario 1: Mass campaign

Under this scenario, three separate targeted campaigns, undertaken each year before the malaria parasite transmission season, are organised to vaccinate children with the first three doses of the vaccine. Children under 5 years of age, who complete the first 3 doses in year one, receive subsequent annual doses (doses 4–7) during the same mass campaign events.

#### Scenario 2: Routine EPI

Under this scenario, all doses are delivered through the existing routine immunisation delivery system. While children are encouraged to receive vaccination right before the transmission season, eligible children are given their vaccination any time throughout the year for the first three doses. Children completing first three doses are given subsequent annual doses also via the routine immunisation delivery system before the transmission season.

#### Scenario 3: Mixed delivery

Under this scenario, the vaccine is delivered using a combination of the mass campaign approach and the existing EPI delivery system. The first three doses are delivered to eligible children through the routine EPI schedule, as in scenario 2. Subsequent annual doses given to children who have completed first three doses are delivered via one targeted mass campaign each year before the transmission season.

### Costing approach

We used an activity-based costing approach where specific activities under each scenario are identified and costed individually by mapping them with the potential resource requirements to generate cost estimates. The level of detail on the delivery strategies and activities, derived through stakeholder consultation, focused on the core components of costing. All activities are grouped into standard categories for the vaccination programme, such as procurement, planning, training, communication, sensitisation, social mobilisation and service delivery, that is, vaccine administration.^9–11^ Within each category, the levels and types of subactivities vary by implementation scenario and country, reflecting needs and current capacities as well as cost consequence. Detailed description of cost categories and subactivities by scenario are included in online supplemental appendix table 1.

10.1136/bmjgh-2022-011316.supp1 Supplementary data

We adapted the Malaria Vaccine Introduction Planning and Costing Tool (MVICT), developed and used previously to estimate the cost of malaria vaccine delivery,^5^ to account for the seasonal nature of the intervention in this analysis. The MVICT is a Microsoft Excel-based costing tool developed by PATH in collaboration with the WHO and Levin & Morgan LLC. The tool estimates the unit cost by activity and subactivities based on user assumptions for any given scenario. The tool can be made available to the readers on request.

Costs were further categorised as introduction (or initial set up) and recurrent costs as well as financial and economic costs.

Introduction costs consist of the value of resources that last longer than 1 year and include costs associated with purchasing capital resources (such as cold chain equipment) as well as non-recurring activities for introduction, such as initial training, social mobilisation and communication material development. Recurrent costs include operational costs of the programme such as the value of procuring vaccines, distribution, monitoring and supervision, personnel time as well as costs of short-term training activities that typically last less than a year.

Financial costs represent direct outlays of resources needed for vaccine delivery and include costs of resources purchased for programme implementation such as injection supplies, outreach allowances and per diem, resources used in training and new communication materials. Economic costs represent the opportunity costs of all resources and include all financial costs plus the value of existing resources within the existing immunisation programme that are used, specifically, salaries of current health personnel for their time used in malaria vaccine delivery as well as donated items. The cost of vaccine doses is excluded from the financial cost and included only in economic cost estimates under the assumption that the necessary vaccine doses would be donated to the government. Costs of other immunisation supplies, as well as vaccine procurement add-on costs (such as shipping and handling), are assumed to be financial costs to the government.

Costs of capital items were annualised over their respective estimated useful life years. Expenditures associated with initial setup were considered capital costs and were annualised and discounted at 3% over an assumed useful life of 5 years.

### Data

Activity maps detailing specific subactivities required for vaccine implementation under each scenario were developed in discussions with the EPI programmes and were guided by recent new vaccine introduction experiences and programme expectations at the time of study conduct (April through December 2021). Key data inputs and assumptions regarding input resource requirements and unit costs (see online supplemental appendix table 2) were collected as part of the costing interviews with in-country stakeholders and administrative/financial record review. These data were informed by the recent new vaccine introduction in each study country.

To inform potential recurrent costs, we collected primary data from representative samples of health administrative units, vaccine stores and health facilities at regional, subregional/district and facility levels in two regions in each country. Districts and health facilities were selected by considering potential differences that are anticipated to drive variation in cost of delivery. See list of districts and health facilities selected for data collection in online supplemental appendix table 3.

Quantities of vaccine and supplies are derived based on the projected birth cohorts/target population adjusted for the anticipated coverage provided by the national immunisation programmes. Any data gaps were supplemented by assumptions guided by country experiences with previous vaccine introductions. The consolidated data on all subactivities and the assumptions on respective input resources and unit costs used to generate the final cost estimates were all validated with the EPI programme representatives in each country. All cost data were collected in the local currency in 2021 units. Cost estimates are reported in both local currency units and in 2021 USD units.

### Capacity consideration and shared input

We estimated the cold chain capacity (volume) requirement based on quantity of RTS, S doses needed and projected the costs of additional cold chain expansion at all levels. In Mali, new capital investments, especially vehicles for the EPI programme, were identified as necessary to fill in health system capacity constraints and were included in the cost estimates. To account for the incremental resource requirements for distribution in a routine setting, inputs shared with the existing system are attributed to RTS, S based on direct allocation (10%). The contribution of vaccinators to the malaria vaccine implementation is estimated based on time required to administer the vaccine under the routine EPI scenario, as reported by health workers during facility surveys. While there is the possibility of coadministered, we do not make specific assumptions on codelivery of RTS, S vaccine with other existing childhood vaccines in EPI schedule as we focus on the costs incremental to the existing vaccines. For a mass campaign, service delivery costs are estimated by accounting for the total number of days for each mass campaign session and the associated resource need. For all other resources, we assume 100% existing spare capacity in the immunisation system to accommodate malaria vaccine introduction and delivery.

### Cost estimates

The key outputs of the analysis are reported as the incremental cost per dose administration, cost of delivery per dose, cost per first three dose completion and cost of delivery per first three dose completion. Annualised cost of the introduction/initial setup costs for the study duration were added to the recurrent costs across all activity categories during the same period to generate the total cost of the programme. The cost per dose administered is calculated by dividing the total cost of the programme by the total number of doses administered throughout the duration of the analysis under a given scenario. The cost of delivery per dose is calculated by subtracting the commodity cost (vaccine and immunisation supplies) and the procurement add-on costs from the total cost and dividing by the total number of doses delivered. Costs per first three dose completion is calculated by dividing the total cost of the programme by the total number of children who receive at least the first three doses of vaccine. Cost of delivery per first three doses completion is calculated by dividing the total cost of the programme net of commodity costs, by the total number of children who receive at least the first three doses of vaccine (see online supplemental appendix table 4 for calculations).

### Sensitivity analysis

Baseline cost estimates are generated using the input values and assumptions in table 1. To understand the implications of input value choices on cost estimates, one-way sensitivity tests are performed for a subset of critical input data, over a range of alternative values, including vaccine price and coverage. Cost estimates under alternate input assumptions are reported separately.

**Table 1:** Key data input and assumptions used in the analysis

Input	Burkina Faso	Mali	Data source
**Vaccination schedule and target population**			
Target age group for vaccination	0–5 years		Assumption supported by clinical trial^2^
Age and timing for first three doses	5–17 months at first vaccination		
Maximum number of doses per child	7		
Number of surviving infants in year 2018	805 961	783 972	Annual statistical yearbooks^13–15^
Population growth rate	3.00%	3.36%	
**Coverage and drop out**			
Coverage, dose 1	100%		Assumptions informed by EPI
Drop out, dose 1 to 2	4%		
Drop out, dose 2 to 3	8%		
Coverage, dose 4–7	80%		
**Vaccine product characteristics**			
Vaccine presentation (dose per vial)	2		GSK^5^
Vaccine packaged volume (cm^3^/dose)	9.2		
Vaccine wastage	10%		Assumed
Injection devices and safety boxes wastage	10%		
Vaccine/injection device and safety boxes (buffer stock)	25%		
Vaccine product cost assumptions (USD)			
Vaccine price per dose	$5 ($2–$10)		Assumed^16^
Cost per injection syringe	$0.20		Assumed, MVIP
Cost per reconstitution syringe	$0.05		
Cost per safety box (100-syringe capacity)	$1.00		
**Procurement add-on charges on as a % of product cost**			
Freight, insurance, inspection	7.60%		Assumed (observed during MVIP in Ghana)
Handling fee	3.00%		
**Service delivery**			
Proportion of children vaccinated in routine outreach sessions	23%	40%	Primary data collected during health facilities survey
Average time spent per vaccination (routine fixed clinic)	10 min	18 min	
Average children vaccinated per campaign site	200	500	Assumed
Average children vaccinated per routine outreach session	50	50	
**Salaries**			
Staff salary per month; range by staff cadre	64 000–5 25 487 CFA	16 377–40,155 CFA	17
Average vaccinators’ salary per month	53 333 CFA	22 778 CFA	
**Others**			
Exchange rate (1USD=)	575.6	575.6	18
Useful life years for introduction activities*	5 years		Assumed
Discount rate	3%		

*Not applicable for recurring activities.

CFA, West African Franc; GSK, GlaxoSmithKline; MOH, Ministry of Health; USD, US dollar.

### Patient public involvement

No patients were involved in this study. We have included roles and relationships between different members of the research team in the reflexivity statement (see online supplemental file).

10.1136/bmjgh-2022-011316.supp2 Supplementary data

## Results

### Target population, projected vaccinations and total costs

Table 2 shows the number of vaccinations throughout the analysis period, 2022–2026. At an assumed coverage level at baseline (same for all scenarios, table 1), about 4.79 million and 4.5 million surviving infants are targeted for vaccination in Mali and Burkina Faso, respectively (table 2).

**Table 2:** Target population, projected vaccinations and total costs (in USD), 2022–2026

Metric	Burkina Faso		Mali	
**Projected outputs**				
Target population for vaccination	4 539 545		4 796 000	
Projected vaccinations	19 132 211		20 186 822	
Projected number of children completing the first three doses	4 009 326		4 235 827	
**Projected costs**	**Financial**	**Economic**	**Financial**	**Economic**
Scenario 1: mass campaign				
Total cost, annualised	29 563 102	145 247 375	49 787 354	171 187 930
Total introduction cost, annualised	2 731 879	3 376 512	6 395 712	7 499 521
Total recurrent cost	26 831 223	141 870 863	43 391 641	163 688 409
Scenario 2: routine EPI				
Total cost, annualised	18 067 278	131 965 973	24 564 962	144 969 203
Total introduction cost, annualised	2 840 869	3 586 112	9 399 259	11 099 610
Total recurrent cost	15 226 408	128 379 861	15 165 703	133 869 594
Scenario 3: mixed delivery				
Total cost, annualised	21 579 227	136 245 677	38 811 304	159 669 492
Total introduction cost, annualised	2 715 720	3 379 695	10 391 365	11 584 202
Total recurrent cost	18 863 507	132 865 983	28 419 938	148 085 290

EPI, Expanded Programme on Immunisation.

Unless otherwise noted, all cost estimates are based on an assumed vaccine price of US$5.00 per dose. The total financial cost (excludes vaccine cost), for the duration of the analysis, is estimated to range from $24.6 to $49.8 million (Mali) and $18.1 to $29.6 million (Burkina Faso) (table 2). The economic cost for the 5-year period ranges from $144.9 to $171.1 million (Mali) and $131.9 to $145.2 million (Burkina Faso). The total programme cost includes annualised introduction costs and annual recurrent costs for a seven-dose RTS, S vaccine schedule (four-dose primary schedule plus three annual doses) for the duration of the analysis.

### Unit cost estimates

Across the three delivery scenarios, the financial cost per dose of vaccine administration to the target population is estimated to range from $1.47 to $2.71 (Mali) and $1.11 to $1.71 (Burkina Faso). The economic cost per dose of vaccine administration ranges from $7.38 to $8.68 (Mali) and $7.04 to $7.73 (Burkina Faso). The cost of delivery per dose, excluding vaccine and other immunisation supplies cost, ranges from $0.76 to $1.99 (Mali) and $0.39 to $0.99 (Burkina Faso), across the three scenarios. The cost per first three dose completion is estimated to range from $5.80 to $11.08 (Mali) and $4.51 to $7.37 (Burkina Faso) (see table 3 and online supplemental appendix figure 1). Across both countries, a mass campaign is the costliest approach to vaccine delivery and the routine EPI delivery is the least costly option.

**Table 3:** Unit cost estimates of seasonal malaria vaccine delivery, by vaccination scenario (in USD)

	Burkina Faso				Mali			
	Cost of delivery per dose*	Cost per dose administered†	Cost of delivery per first three doses completion‡	Cost per first three doses completion§	Cost of delivery per dose*	Cost per dose administered†	Cost of delivery per first three doses completion‡	Cost per first three doses completion§
**Financial cost**								
Mass campaign	$0.99	$1.71	$3.97	$7.37	$1.99	$2.71	$7.68	$11.08
Routine EPI	$0.39	$1.11	$1.10	$4.51	$0.76	$1.47	$2.40	$5.80
Mixed delivery	$0.58	$1.29	$1.98	$5.38	$1.28	$2.00	$5.76	$9.16
**Economic cost**								
Mass campaign	$1.17	$7.73	$4.94	$36.23	$2.12	$8.68	$9.16	$40.41
Routine EPI	$0.48	$7.04	$1.62	$32.91	$0.82	$7.38	$2.97	$34.22
Mixed delivery	$0.70	$7.26	$2.69	$33.98	$1.37	$7.93	$6.44	$37.69

*The cost of delivery per dose is calculated by subtracting the commodity cost (vaccine and immunisation supplies) and the procurement add-on costs from the total cost and dividing by the total number of doses delivered.

†Cost per dose administered is calculated by dividing the total cost of the programme by the total number of doses administered throughout the duration of the analysis under a given scenario.

‡Cost of delivery per first three doses completion is calculated by dividing the total cost of the programme net of commodity costs, by the total number of children who receive at least the first 3 doses of vaccine.

§Cost per first three dose completion is calculated by dividing the total cost of the programme by the total number of children who receive at least the first three doses of vaccine. See online supplemental appendix table 4 for calculations.

EPI, Expanded Programme on Immunisation.

### Cost drivers

Recurrent costs constitute the major share of the total costs across all scenarios (online supplemental appendix figure 2). Introduction/initial setup costs constitute between 9.2% and 12.8% of financial cost and 2.3% and 4.4% of economic cost, across the two countries. Introduction cost share is relatively higher for financial cost under the EPI approach (scenario 2), which ranges between 16% and 38% of the total cost across the two countries.

The cost of vaccine procurement add-on and immunisation commodities constitutes the largest driver of financial cost across all scenarios. Service delivery under mass campaign constitutes 27% and 32% of the total financial cost in Mali and Burkina Faso, respectively. Under the mixed delivery scenario, service delivery accounts for between 8% and 10% of total financial cost. Excluding commodity cost, service delivery is the main cost driver for mass campaign delivery accounting for 36% and 55% of financial cost in Mali and Burkina Faso, respectively. The financial cost share of service delivery ranges between 2% and 8% under routine EPI and between 12% and 23% for the mixed delivery scenario. For economic costs, service delivery is the main cost driver under mass campaign delivery, accounting for up to 57% of the cost, whereas the economic cost share for service delivery ranges between 6% and 16% under routine EPI and between 14% and 27% under mixed delivery. The distribution of resource requirements, as a proportion of total costs, for each scenario is provided in online supplemental appendix Tables 5A–C and 6A–C.

### Sensitivity analysis

The analysis shows that the unit cost estimates are most sensitive to vaccine price, and its impact is most substantial on cost per first three dose completion and economic cost for all scenarios. Overall, across the two countries under different coverage and vaccine price assumptions, the financial cost per dose administered ranges between $1.33 and $3.99 (mass campaign), $0.73 and $2.89 (routine EPI) and $0.92 and $3.12 (mixed delivery). The economic cost per dose administered ranges between $3.85 and $15.14 (mass campaign), $3.16 and $13.84 (routine EPI) and $3.38 and $14.40 (mixed delivery). Across the two countries, under different coverage assumptions, the financial cost of delivery per dose ranges between $0.91 and $3.28 (mass campaign), $0.37 and $2.18 (routine EPI) and $0.54 and $2.41 (mixed delivery), and the economic cost of delivery per dose ranges between $1.08 and $3.84 (mass campaign), $0.45 and $2.30 (routine EPI) and $0.66 and $2.56 (mixed delivery). The range of cost estimates under alternative coverage and vaccine price assumptions is in table 4 and online supplemental appendix table 7.

**Table 4:** Unit cost estimates (in USD) at various coverage assumptions

Metric	Coverage	Sn1: Mass campaign		Sn2: Routine EPI		Sn3: Mixed delivery	
		Financial	Economic	Financial	Economic	Financial	Economic
**Mali**							
Cost per dose administered	High (100%)	2.71	8.86	1.47	7.38	2.00	7.93
Cost per dose administered	Medium (70%)	3.26	9.26	1.79	7.71	2.48	8.44
Cost per dose administered	Low (50%)	3.99	10.04	2.22	8.15	3.12	9.11
Cost per dose administered	EPI anticipated*	3.29	9.27	2.89	8.92	3.02	8.89
Cost of delivery per dose	High (100%)	1.99	2.12	0.76	0.82	1.28	1.37
Cost of delivery per dose	Medium (70%)	2.54	2.7	1.08	1.15	1.77	1.88
Cost of delivery per dose	Low (50%)	3.28	3.48	1.5	1.59	2.41	2.56
Cost of delivery per dose	EPI anticipated*	2.58	2.74	2.18	2.3	2.31	2.46
Cost per first three doses completion	High (100%)	11.08	40.41	5.8	34.22	9.16	37.69
Cost per first three doses completion	Medium (70%)	13.88	42.8	6.79	35.39	11.31	40.06
Cost per first three doses completion	Low (50%)	16.71	45.98	8.11	36.95	14.18	43.22
Cost per first three doses completion	EPI anticipated*	13.04	39.67	8.92	34.3	12.29	38.07
Cost of delivery per first three doses completion	High (100%)	7.68	9.16	2.4	2.97	5.76	6.44
Cost of delivery per first three doses completion	Medium (70%)	10.48	11.54	3.39	4.13	7.91	8.8
Cost of delivery per first three doses completion	Low (50%)	13.31	14.72	4.71	5.69	10.78	11.96
Cost of delivery per first three doses completion	EPI anticipated*	9.91	10.89	5.97	7.18	9.27	10.3
**Burkina Faso**							
Cost per dose administered	High (100%)	1.71	7.73	1.11	7.04	1.29	7.26
Cost per dose administered	Medium (70%)	1.9	7.96	1.26	7.21	1.48	7.48
Cost per dose administered	Low (50%)	2.16	8.24	1.47	7.44	1.73	7.77
Cost per dose administered	EPI anticipated*	1.63	7.65	1.08	7.01	1.26	7.23
Cost of delivery per dose	High (100%)	0.99	1.17	0.39	0.48	0.58	0.7
Cost of delivery per dose	Medium (70%)	1.19	1.39	0.55	0.65	0.77	0.92
Cost of delivery per dose	Low (50%)	1.44	1.68	0.75	0.88	1.02	1.21
Cost of delivery per dose	EPI anticipated*	0.91	1.08	0.37	0.45	0.54	0.66
Cost per first three doses completion	High (100%)	7.37	36.23	4.51	32.91	5.38	33.98
Cost per first three doses completion	Medium (70%)	7.96	36.98	4.92	33.46	5.96	34.73
Cost per first three doses completion	Low (50%)	8.75	37.97	5.47	34.19	6.73	35.75
Cost per first three doses completion	EPI anticipated*	7.11	36.09	4.5	33.29	5.34	34.31
Cost of delivery per first three doses completion	High (100%)	3.97	4.94	1.1	1.62	1.98	2.69
Cost of delivery per first three doses completion	Medium (70%)	4.56	5.68	1.51	2.17	2.55	3.45
Cost of delivery per first three doses completion	Low (50%)	5.34	6.68	2.06	2.9	3.32	4.46
Cost of delivery per first three doses completion	EPI anticipated*	3.68	4.58	1.05	1.55	1.89	2.57

*EPI anticipated coverage was very high for dose 1 (100% for all scenarios in Burkina Faso but lower for Mali (Mass campaign: 90%, Routine EPI: 50% Mixed delivery: 70%). Coverage values in column ‘coverage’ is for dose 1. For the other doses, dropout rates used in the primary analysis (given in table 1) are used.

EPI, Expanded Programme on Immunisation.

## Discussion

Seasonal vaccine delivery is uncommon especially in low-and middle-income countries (LMICs). The routine immunisation programme needs several adaptations to be able to accommodate seasonal vaccine delivery and achieve health impact. Necessary adaptations may include additional training activities and guidance to staff, increased human resource mobilisation in season, community mobilisation and sensitisation activities to encourage seasonal utilisation using mass vaccination campaign methodologies. These adaptations have cost consequences to the respective programmes. Several studies assessing costs of malaria vaccine delivery are available,^5–7^ yet all of these evaluate costs in the context of routine immunisation. Seasonal vaccine delivery cost estimates are almost non-existent, especially in the LMICs. Our study fills a knowledge gap on the economic implications of seasonal vaccine planning and effective decision-making around delivery approaches and resource mobilisation.

Our findings suggest that vaccine delivery using a mass campaign approach is most costly compared with other delivery approaches. Across the two countries, the economic cost per dose administered ranges between $7.73 and $8.68 (mass campaign), $7.04 and $7.38 (routine EPI) and $7.26 and $7.93 (mixed delivery). The financial cost per dose administered ranges between $1.71 and $2.71 (mass campaign), $1.11 and $1.47 (routine EPI) and $1.29 and $2.00 (mixed delivery). Administering malaria vaccine under the routine EPI scenario is the least costly option as this approach requires the least amount of adaptation to existing immunisation programmes. Under a mass campaign approach, three targeted mass campaigns would occur each year before the transmission season, which would require significantly different levels of effort and types of activities to prepare for and complete compared with the routine EPI approach (see online supplemental appendix table 1 for detailed description of subactivities across scenarios). For example, under the mass campaign approach, service delivery, which requires human resource mobilisation necessitating direct allowances for both staff and volunteers, is one of the major cost drivers accounting for between 36% and 55% of total financial cost (excluding commodity cost). On the other hand, service delivery (excluding commodity cost) accounts for 2%–8% and 12%–23% of financial cost under the routine EPI and mixed delivery scenarios, respectively. This corroborates the finding that mass campaign delivery, in general, is more costly at a given coverage level as it requires mobilisation of a large group of human resources,^12^ often diverting them away from routine health service delivery along with other resources. Mass campaigns, nonetheless, have capacity to achieve higher coverage than the routine immunisation scenario, which is not directly differentiated in this analysis.

In Mali, for the mass campaign approach, stakeholders indicated that an initial training for health workers when the vaccine is introduced, as well as annual refresher trainings before each campaign season, would be necessary activities. As a result, the financial cost for training was about 2.5 times higher under the mass campaign approach compared with the routine EPI approach in that country. Also, vaccine distribution costs during repeated campaigns added substantially to the total financial cost for mass campaigns, which was not the case for the routine EPI approach, which leverages existing vaccine distribution channels. The financial cost of vaccine distribution was between 1.5 and 2 times higher for the mixed delivery approach and between 3 and 4 times higher in the mass campaign approach, compared with the routine EPI approach. Furthermore, under the mass campaign approach, most planning and coordination, and sensitisation and social mobilisation subactivities, are identified as necessary each year, rendering higher recurring costs for these categories under the mass campaign approach. On the other hand, most planning and social mobilisation activities were concentrated in year 1 only under the routine EPI approach, and in years 1 and 2 only in the mixed delivery approach. As a result, the total cost for planning and coordination is more than double for the mass campaign approach compared with the routine EPI approach and even higher as compared with the mixed delivery approach, particularly in Burkina Faso. In Mali, the differences in total cost were about five times higher for both the mass campaign and mixed delivery approach compared with the routine EPI scenario. The differences in cost estimates between the two countries partly reflect the differences in input prices, including staff salaries, among others.

Cost estimates are sensitive to underlying assumptions, most profoundly the vaccine price, as shown in other similar studies.^5–7^ Given malaria vaccines were not yet recommended for broader use at the time of analysis, uncertainty around various parameters is evident. Amid uncertainties in implementing modalities at the time of study conduct, potential activities for programme introduction and delivery for each alternative scenario of programme delivery were extensively discussed and identified together with in-country stakeholders to ensure the robustness of the cost estimates. However, as the malaria vaccine product was not yet recommended for broader use, these discussions with in-country stakeholders, particularly around scenarios synthesis and prioritisation of feasible delivery scenarios, were challenging, as there were uncertainties with regards to delivery approaches and difficulties in articulating activities to fit in with possible future needs. Due to this, only the core components of the strategies were discussed and included in the study. The assumptions made on delivery scenarios as well as input resources are reliant on the understanding of the discussants. The assumptions were informed by significant deliberation and discussion and draw on previous new vaccine introductions using different delivery approaches including mass campaigns.

A few studies have estimated that the economic cost of delivering RTS, S under routine EPI in Burkina Faso along with a few other sub-Saharan Africa countries. Galactionova *et al*^6^ reported the economic cost of delivery per dose (net of commodities) in Burkina Faso at $0.72. Sicuri *et al*^7^ reported the cost per fully vaccinating a child with four-dose schedule using existing EPI platform to be $2.58. Our results suggest that under the routine EPI delivery, the cost per first three dose completion in Burkina Faso is $1.62. While the cost estimates are not directly comparable across these studies due to differences in underlying assumptions and cost calculations, estimates in the current analysis for Burkina Faso are broadly aligned with the reported range.

Cost estimates generated from this analysis provide useful insights into the cost drivers of alternative delivery approaches and can be applicable not just to RTS, S but also to other vaccines being considered for seasonal delivery. While the estimates are generated for two countries in the Africa Sahel region with largely similar socioeconomic profile, the cross-country cost estimates provide a range that may be used by other countries, not included in the study, to estimate costs. However, we caution against making extensive comparisons cross-country, as we used a country-specific activity-based costing approach reflecting specific inputs and assumptions agreed on by each country team at the time of analysis.

Our study has some limitations. Cost projections are largely dependent on the most feasible strategy chosen for costing by country and is likely to be context specific, limiting the generalisability of the results outside of the study countries. Another potential limitation is the baseline assumption that the same level of vaccine coverage will be achieved across all delivery scenarios. This may not be true as mass campaigns, due to their targeted nature, are likely to achieve higher coverage as they bring vaccine delivery closer to the communities, provide targeted mobilisation of human resources including volunteers for vaccine administration and promote strong community mobilisation, which may not be consistent with routine EPI delivery approaches. In the mixed delivery approach, the one targeted mass campaign could likely lead to higher coverage for the subsequent annual doses as well as catch up children who missed vaccinations via routine EPI administration. Given the lack of knowledge a priori around these parameters, we look at the impact of different coverage levels on the unit cost estimates, which provides useful lower and upper bound estimates under possible coverage ranges. The analysis, however, does not account for the extra effort to increase coverage and only reflects a change in the denominator. The analysis also does not differentiate vaccine wastage rates across the scenarios, though one can expect to see variation in vaccine wastage rate by delivery scenario. Nonetheless, the non-commodity cost of vaccine delivery generated in the analysis reflects the recurring cost to the programme, unaffected by some of the key unknown parameters. The prospective cost projections in this study builds on many assumptions, while informed and validated by the EPI programmes in respective counties based on their previous new vaccine introduction experiences, would need further validation with actual costs of implementation when the RTS, S vaccine is deployed in seasonal settings.

The WHO recommends that countries consider a five-dose malaria vaccine strategy in settings of highly seasonal malaria transmission.^3^ The seven-dose schedule considered in this study was guided by the ongoing clinical trial in these countries, looking at RTS, S vaccination alongside administration of SMC.^2^ While the timing of seasonal delivery of these interventions (RTS, S vaccination at or before the transmission season and SMC administration during the transmission season) may preclude direct coadministration of these two interventions, there might be some room for efficiency in coplanning and delivery, which is not investigated in this study. The definition of a fully immunised child (FIC) in the current context is not clear, and, because of this, we did not estimate the cost per FIC, opting instead to estimate the cost per first three dose completion.

Implementation feasibility and affordability are critical input in making decisions about whether or not to adopt new vaccines and also around planning for sustainable vaccine delivery. While cost of implementation is one of the critical components to assess feasibility and affordability of any new vaccine, the effectiveness of the delivery approach in achieving the desired level of coverage is also critical. Health impact is achieved through coverage, and this study does not address potential differences in coverage by delivery strategy. Therefore, it cannot inform the relative value of competing delivery strategies based on cost alone. This remains an important area for future investigation. Despite some limitations, this study provides useful information for decision-makers on the potential cost differences associated with several seasonal malaria vaccine delivery strategies.

## Data Availability

All data relevant to the study are included in the article or uploaded as supplementary information.
