# Supplementary material for: Cost of introducing and delivering malaria vaccine (RTS, S/AS01E) in areas of seasonal malaria transmission, Mali and Burkina Faso
Source: BMJ Glob Health. 2023 Apr 17;8(4):e011316. doi: 10.1136/bmjgh-2022-011316 (PMC10111920; doi:10.1136/bmjgh-2022-011316)
Supplement: Supplementary data [file bmjgh-2022-011316supp001.pdf]

Appendices

Appendix Table 1.a: List of sub-activities by cost category and activity occurrence frequency in Mali

Activities	Scenario 1: Mass campaign	Scenario 2: Routine EPI	Scenario 3: Mixed delivery
**Vaccine and injection supplies procurement**			
Procurement of vaccine	Each year	Each year	Each year
Procurement of supplies- 2ml syringe	Each year	Each year	Each year
Procurement of supplies- RUP syringe	Each year	Each year	Each year
Procurement of supplies- Safety boxes	Each year	Each year	Each year
**Distribution**			
Receive vaccine and supplies at the national store	Three times each year	Quarterly (4 times) a year, shared cost	Quarterly (4 times) a year, shared cost, plus one time for campaign starting year 2
Distribution of vaccine and supplies from national store to regional (up to district) stores	Three times each year	Quarterly (4 times) a year, shared cost	Quarterly (4 times) a year, shared cost, plus one time for campaign starting year 2
Collection of vaccine and supplies from regional store by District stores	NA	Monthly, shared cost	Monthly, shared cost
Distribution of vaccine and supplies from district store to health facilities	Three times each year	NA	One time each year
Collection of vaccine and supplies from district store by health facilities	NA	Monthly, shared cost	Monthly, shared cost
**Planning and coordination**			
Planning for introduction at National level	Once in year 1 only	Once in year 1 only	Once in years 1 and 2 only
Develop plans for introduction/campaign	Once in year 1 only	Once in year 1 only	Once in years 1 and 2 only
National level macroplanning for introduction/campaign	Once each year	Once in year 1 only	Once each year
Regional level microplanning for introduction/campaign	Once each year	Once in year 1 only	Once each year
District level microplanning for introduction/campaign	Once each year	Once in year 1 only	Once each year
Health facility level microplanning for introduction/campaign	Once each year	Once in year 1 only	Once each year
**Training**			
Develop training materials and tools and TOT at national level	Once in year 1 only	Once in year 1 only	Once in year 1 only

Diawara H, et al. BMJ Global Health 2023; 8:e011316. doi: 10.1136/bmjgh-2022-011316

Activities	Scenario 1: Mass campaign	Scenario 2: Routine EPI	Scenario 3: Mixed delivery
Refresher Training of trainers for the national level staff	Once each year from year 2	NA	Once each year from year 2
Training for the regional level staff	Once in year 1 only	Once in year 1 only	Once in year 1 only
Refresher training at regional level	Once each year from year 2	NA	Once each year from year 2
Training for the district level staff	Once in year 1 only	Once in year 1 only	Once in year 1 only
Refresher training for the district level staff	Once each year from year 2	NA	Once each year from year 2
Training for the health facility staff	Once in year 1 only	Once in year 1 only	Once in year 1 only
Refresher training for the health facility staff	Once each year from year 2	NA	Once each year from year 2
Training of community health workers	Once each year	Year 1 only	Once each year
**Communication (IEC)**			
Production of communication materials (Sketch, spot, trailers)	Once in year 1 only	Once in year 1 only	Once in year 1 only
Broadcasting of communication material in National level (TV/Radio)	Each year	Year 1 only	Each year
Printing of IEC materials	Once in year 1 only	Once in year 1 only	Once in year 1 only
Broadcasting of communication material in local (district) level (TV/Radio)	Each year	Year 1 only	Each year
**Social mobilization**			
Organize press conference for introduction	Once in year 1 only	Once in year 1 only	Once in year 1 only
National level campaign launch	Once each year. Full cost for first 2 years, campaign scale halved for other years.	Once in year 1 only	Once each year. Full cost for first 2 years, campaign scale halved for other years.
Coordination and preparation of community information session by Social Development Unit	Once each year	Once in year 1 only	Once each year
Hold information sharing/launch session in district	Each year	Years 1 and 2 only	Each year
Community sensitization/mobilization by community health volunteers	Each year	Years 1 and 2 only	Each year
**Monitoring and evaluation**			
Post introduction/campaign evaluation	Year 1 only	Year 1 only	Year 1 only
**Supervision**			
Introduction supervision by a team of national, regional and district staff	Three times each year	Two times each year in years 1 and 2 only	Two times each year

Activities	Scenario 1: Mass campaign	Scenario 2: Routine EPI	Scenario 3: Mixed delivery
Introduction supervision by health facility staff	Three times each year	Two times each year in years 1 and 2 only	Two times each year
Routine quarterly supervision by a team of national, regional and district staff	NA	Quarterly (4 times) each year, shared cost	Quarterly (4 times) each year, shared cost
Routine quarterly supervision by regional level staff	NA	Quarterly (4 times) each year, shared cost	Quarterly (4 times) each year, shared cost
Routine quarterly supervision by district level staff	NA	Quarterly (4 times) each year, shared cost	Quarterly (4 times) each year, shared cost
**Service delivery**			
Vaccination administration through routine EPI clinic	NA	Each year	Each year
Vaccination administration through routine EPI outreach	NA	Each year	Each year
Vaccination administration during seasonal campaign	Three times each year	NA	One time each year starting year 2
**Cold chain procurement**			
Cold room walk in (capacity: 30 cu m) added at national level	Years 1, 3, and 5	Years 1, 3, and 5	Years 1, 3, and 5
Cold room walk in (capacity: 10 cu m) added to 11 regional levels	Years 1, and 3	Years 1, and 3	Years 1, and 3
Refrigerators (capacity: 145,000 cu cm) added to 75 district levels	Years 1, and 3	Years 1, and 3	Years 1, and 3
Cold boxes added to each health facility (5 cold boxes per health facility	Years 1, and 3	Years 1, and 3	Years 1, and 3
Maintenance of cold chain	Each year, shared cost	Each year, shared cost	Each year, shared cost
**Other capital equipment purchase**			
Vehicles for national level to support implementation	8 vehicles in year 1	8 vehicles in year 1	8 vehicles in year 1
Vehicles for regional level to support implementation	2 per implementing region in year 1	2 per implementing region in year 1	2 per implementing region in year 1
Vehicles for district level to support implementation	2 per implementing district in year 1	2 per implementing district in year 1	2 per implementing district in year 1

Appendix Table 1.b: List of sub-activities by cost category and activity occurrence frequency in Burkina Faso

Activities	Scenario 1: Campaign	Scenario 2: Routine EPI	Scenario 3: Mixed delivery
**Vaccine and injection supplies procurement**			
Procurement of vaccine	Each year	Each year	Each year
Procurement of supplies- 2ml syringe	Each year	Each year	Each year
Procurement of supplies- RUP syringe	Each year	Each year	Each year
Procurement of supplies- Safety boxes	Each year	Each year	Each year
**Distribution**			
Receive vaccine and supplies at the national store	Three times each year	Quarterly (4 times) a year, shared cost	Quarterly (4 times) a year, shared cost, plus one time for campaign starting year 2
Distribution of vaccine and supplies from national store to regional stores	Three times each year	Quarterly (4 times) a year, shared cost	Quarterly (4 times) a year, shared cost, plus one time for campaign starting year 2
Collection of vaccine and supplies from regional store by District stores	Three times each year	Bi-monthly (6 times) a year, shared cost	Bi-monthly (6 times) a year, shared cost
Distribution of vaccine and supplies from district store to health facilities	Three times each year	NA	One time each year starting year 2
Collection of vaccine and supplies from district store by health facilities	NA	Bi-monthly (6 times) a year, shared cost	Bi-monthly (6 times) a year, shared cost
**Planning and coordination**			
Develop plans for introduction/campaign	Three events in year 1 only	Three events in year 1 only	Three events in year 1 only
Planning for introduction at national level for campaign	Once each year	NA	Once each year starting year 2
Regional level microplanning for introduction/campaign	Once each year	Once in year 1 only	Once each year
District level microplanning for introduction/campaign	Once each year	Once in year 1 only	Once each year
Health facility level microplanning for introduction/campaign	Once each year	Once in year 1 only	Once each year
Routine microplanning at national, regional, district, and health facility levels	NA	Once each year, shared cost	Once each year, shared cost
**Training**			
Develop training materials and tools	Once in year 1 only	Once in year 1 only	Once in year 1 only
Training of trainers at the national level	Once in year 1 only	Once in year 1 only	Once in year 1 only

Activities	Scenario 1: Campaign	Scenario 2: Routine EPI	Scenario 3: Mixed delivery
Training at the regional level	Once in year 1 only	Once in year 1 only	Once in year 1 only
Training at district levels	Once in year 1 only	Once in year 1 only	Once in year 1 only
Training at the health facility level	Once in year 1 only	Once in year 1 only	Once in year 1 only
Training of community health workers/volunteers for campaign	Once in year 1 only	NA	Once in year 1 only
**Initial sensitization**			
Stakeholder sensitization at national level	Once in year 1 only	Once in year 1 only	Once in year 1 and 2 only
Sensitization of media personnel at national level	Once in year 1 only	Once in year 1 only	Once in year 1 and 2 only
Stakeholder sensitization at regional level	Once in year 1 only	Once in year 1 only	Once in year 1 and 2 only
Stakeholder sensitization at district level	Once in year 1 only	Once in year 1 only	Once in year 1 and 2 only
Stakeholder sensitization at health facility level	Once in year 1 only	Once in year 1 only	Once in year 1 and 2 only
**Communication**			
Development and production of communication materials (Flip charts, posters)	Once in year 1 only	Once in year 1 only	Once in year 1 only
Develop radio/TV message, newspaper spots etc.	Once each year	Once in year 1 only	Once each year
Broadcasting of TV/radio messages, and new paper messages	Once each year	Once in year 1 only	Once each year
Organize media campaigns at regional level	Once each year	Once in year 1 only	Once each year
Organize media campaigns at district level	Once each year	Once in year 1 only	Once each year
**Social mobilization**			
Launch event at national level	Once in year 1 only	Once in year 1 only	Once in year 1 and 2 only
Launch event at regional level	Once in year 1 only	Once in year 1 only	Once in year 1 and 2 only
Organize awareness raising by town criers and mobilizing relays in the villages	Each year, integrated activity- no additional cost	Each year, integrated activity- no additional cost	Each year, integrated activity- no additional cost
Carry out educational talks in health facilities	Each year, integrated activity- no additional cost	Each year, integrated activity- no additional cost	Each year, integrated activity- no additional cost
**Monitoring and evaluation**			
Printing and distribution of monitoring and evaluation tools	Each year	Each year	Each year
Conduct pre and post coverage surveys	Year 2 only	Year 2 only	Year 2 only

Activities	Scenario 1: Campaign	Scenario 2: Routine EPI	Scenario 3: Mixed delivery
**Supervision**			
Implementation (introduction and post introduction) supervision at regional level	Three times each year	Two times in years 1 only	Two times in year 1 and one time each in other years
Implementation (introduction and post introduction) supervision at district level	Three times each year	Two times in years 1 only	Two times each year
Implementation (introduction and post introduction) supervision at health facility level	Three times each year	Two times in years 1 only	Two times each year
Routine quarterly supervision by EPI program management	NA	Four times each year, shared cost	Four times each year, shared cost
Routine semi-annual supervision of immunization program by regional level staff	NA	Two times each year, shared cost	Two times each year, shared cost
Routine semi-annual supportive supervision by district level staff	NA	Two times each year, shared cost	Two times each year, shared cost
**Service delivery**			
Vaccination administration through routine EPI clinic (fixed strategy)	NA	Each year	Each year
Vaccination administration through routine EPI outreach (advanced strategy)	NA	Each year	Each year
Vaccination administration during seasonal campaign	Three times each year	NA	One time each year starting year 2
**Cold chain procurement**			
Cold room walk in (capacity: 30 cu m) added at national level	Years 1, 3, and 5	Years 1, 3, and 5	Years 1, 3, and 5
Cold room walk in (capacity: 10 cu m) added to 11 regional levels	Years 1, and 3	Years 1, and 3	Years 1, and 3
Refrigerators (capacity: 145,000 cu cm) added to 75 district levels	Years 1, and 3	Years 1, and 3	Years 1, and 3
Cold boxes added to each health facility (5 cold boxes per health facility)	Years 1, and 3	Years 1, and 3	Years 1, and 3
Maintenance of cold chain	Each year, shared cost	Each year, shared cost	Each year, shared cost
**Other capital equipment purchase**			
Vehicles purchase to support implementation	NA	NA	NA

Appendix Table 2: List of unit costs used in the analysis
Mali

Items	Units	Unit price (LCU)
Allowance and per diems[1]		
Per diem for non-resident, participant, National level	Per person per day	15,000
Per diem for non-resident, facilitator National level	Per person per day	45,000
Per diem for non-resident, participant, Regional level	Per person per day	5,000
Per diem for non-resident, facilitator Regional level	Per person per day	35,000
Per diem for non-resident, participant, District level	Per person per day	5,000
Per diem for non-resident, facilitator District level	Per person per day	15,000
Allowance for community mobilization, leaders, National level	Per person per day	35,000
Allowance for community mobilization, leaders, Regional level	Per person per day	35,000
Allowance for community mobilization, leaders, District level	Per person per day	35,000
Allowance for community mobilization, participants, all level	Per person per day	5,000
Allowance for vaccine loaders	Per person per day	10,000
Transportation allowance for staff, average	Per person, both ways	5,000
Transportation allowance for staff, long distance	Per person, both ways	30,000
Transportation allowance for non-staff, average	Per person, both ways	5,000
Transportation allowance for non-staff, long distance	Per person, both ways	12,500
Community volunteers' allowance	Per person per day	3,000
Master of Ceremony for National launch event	Per person per day	150,000
Allowance for Hostesses for National launch event	Per person per day	15,000
Supplies and consumables[2]		
Photocopy ink	Each	80,000
Printing paper rim	Each	5,000
Report printing	Each	15,000
Pen	Each	100
Notepad	Each	1,500
Large paper rolls	Each	10,000
Markers	Each	1,000
Stationery package for training, National level	Each	2,500
Stationery package for training, Health facility level	Each	1,500
Drawing and recording materials	Per training	50,000
Drawing of sample and acquisition of SE maps	Per campaign planning event	6,000
Trailer/Spot/Sketch translation fees	Translation in different languages	20,000
Production of skit/sketch for Television	Production fees	2,000,000
Production of Spot/trailer for Television	Production fees	200,000
Production of Spot/trailer for Radio	Production fees	200,000
Broadcasting of trailer/spots in Television	Cost for one broadcast in Television	75,000
Broadcasting of skit/sketch in Television	Cost per show	200,000

Broadcasting of spots/trailer in Radio	Cost per airing	50,000
Newspaper inserts	Cost per insert	75,000
Tarpaulin banners production	Cost per banner	60,000
Printing leaflet (small)	Cost of printing one leaflet	200
Printing leaflet (large)	Cost of printing one leaflet	1,500
Printing poster	Cost pf printing one poster	20,000
Invitation card for program launch and social mobilization	Cost of printing one invitation card	1,000
Printing manuals	Cost of printing	5,000
Vaccination register	Per unit	2,424
Tally register	Per unit	4,848
Monthly vaccination report	Per unit	2,424
Cotton	Per unit	4
Designer contract for developing communication material	Per unit	200,000
Coordination of community information activity	Flat cost at National level	75,000
Hold launches and information sharing session	Flat cost per district	250,000
Printing T-shirt and caps for social mobilization	Cost per unit	200
Other direct costs		
Hall/venue rental, large size, National level	Per day	1,000,000
Hall/venue rental, medium size, National level	Per day	500,000
Hall/venue rental, small size, National level	Per day	250,000
Hall/venue rental, large size, Regional level	Per day	375,000
Hall/venue rental, medium size, Regional level	Per day	200,000
Hall/venue rental, small size, Regional level	Per day	75,000
Hall/venue rental, large size, District level	Per day	150,000
Hall/venue rental, medium size, District level	Per day	75,000
Hall/venue rental, small size, District level	Per day	75,000
Refreshments (coffee, snacks)	Per person per day	1,500
Lunch	Per person per day	2,500
Dinner	Per person per day	2,500
Vehicle hire, small size (Car)	Per vehicle per day	50,000
Vehicle hire, medium size (Vans)	Per vehicle per day	75,000
Vehicle hire, large size (truck)	Per vehicle per day	200,000
Fuel cost	Per liter	660
Trampuline rental	Cost per day	20,000
Sound equipment rental	Cost per day	100,000
Animation artist service contract	Per unit	250,000
Media coverage at National level event	Cost per day	5,000
Chairs rental- metal	Cost per day	100
Chairs rental- plastic	Cost per day	200
Communication costs reimbursement	Flat cost per person	20,000
Average toll/peage	Per trip	15,750
Cold chain and equipment [3, 4]	**Capacity cm3**	

Cold room, walk-in type,10 m³	10,000,000	15,100
Cold room, walk-in type,30 m³	30,000,000	22,500
Cold room, walk-in type,40 m³	40,000,000	27,000
Mains Ref Sure Chill GVR50AC E003/046	46,500	1,642
Mains Ref Sure Chill GVR100AC-M1 E003/047	99,000	2,355
Cold box, RCW 12/CF,PQS E004/004	7,000	614
Mains Ref. Vestfrost VLS400A AC E003/065	145,000	1,322
Mains Ref Aucma MetafridgeCFD50 E003/079	50,000	1,400
Mains Ref Dulas VC225ILR E003/072	184,000	3,318
Mains Ref. Vestfrost VLS400A AC E003/065	145,000	1,322
Mains Ref Aucma MetafridgeCFD50 E003/079	50,000	1,400
Mains Ref SurChl GVR99Lite AC E003/082	98,500	1,200
Mains Ref SureChill GVR225AC-M1 E003/083	225,000	1,910
TCW 2000 AC	60,000	3,034
TCW 3000 AC	150,000	4,346
Cold Box, BlowKing CB/20-CF	20,000	200
TCW15SDD	16,000	4,584
VLS200A	60,000	818
VLS154	170,000	3,140
TCW4000AC	240,000	3,896
VLS400A	145,000	1,118

Sources:

[1] MOH/EPI

[2] Previous new vaccine introduction activity reports from EPI

[3] UNICEF Supply Catalogue (last checked Jan 2018)

[4] Assumptions informed by Kenya Malaria vaccine implementation program costs

Burkina Faso

Items	Units	Unit price (LCU)
Allowance and per diems[1]		
Per diem for resident, Directors	Per person per day	20,000
Per diem for non-resident, Directors	Per person per day	30,000
Per diem for resident, Senior/mid-level Officers	Per person per day	10,000
Per diem for non-resident, Senior/mid-level Officers	Per person per day	27,000
Per diem for resident, administrative support/Drivers	Per person per day	10,000
Per diem for non-resident, administrative support/Drivers	Per person per day	20,000
Per diem for resident, Health facility staff	Per person per day	5,000
Per diem for non-resident, Health facility staff	Per person per day	10,000
Allowance for material handlers, National level	Per person per day	5,000
Allowance for material handlers, District level	Per person per day	2,000
Per diem for resident, non-staff	Per person per day	10,000
Per diem for non-resident, non-staff	Per person per day	27,000

Transportation allowance for non-staff	Per person, both ways	5,000
Lunch allowance - District level	Per person per day	1,500
Lunch allowance - Health facility level	Per person per day	1,000
Per diem during campaign, Health facility staff	Per person per day	5,000
Supplies and consumables[2]		
Notebook	Per piece	1,000
Pen	Per piece	100
Ink (printer cartridge)	Per piece	65,000
Printing paper	Per piece	4,000
Folders	Per piece	1,000
Stationery package for training, Health facility level	Per unit	1,000
Stationery package for training, National level	Per unit	2,500
Printing poster/vaccine calendar	Cost per unit	3,000
Monthly reporting tools	Cost per unit	710
Purchase order form for vaccine	Cost per unit	3,600
Monitoring graphic	Cost per unit	380
Tally sheet	Cost per unit	40
Register for consumables management	Cost per unit	4,300
Register for vaccine management	Cost per unit	5,950
Vaccination card	Cost per unit	15
Plaidoyer (Advocacy) - Flat cost per region	Flat cost per region	400,000
Plaidoyer (Advocacy) - Flat cost per District	Flat cost per district	200,000
Community sensitization - Flat cost per Health Facility	Flat cost per health facility	7,500
Launch event at National level - Flat cost	Flat cost at National level	3,000,000
Launch event at Regional level - Flat cost	Flat cost at Regional level	300,000
Post introduction survey	Flat cost-no detail available	60,000,000
Development of TV spots	Cost per unit paid to media	625,000
Development of radio spots	Cost per unit paid to media	85,000
Broadcasting messages in TV	Cost per broadcast	110,000
Broadcasting messages in radio	Cost per broadcast	8,000
Media coverage - Newspaper Interview (Midi magazine)	Per unit	300,000
Media coverage - at National level	Per unit	250,000
Media coverage TV-Radio program at Regional level	Per unit	100,000
Media coverage TV-Radio program at District level	Per unit	100,000
Other direct costs[2]		
Hall/venue rental, large size, National level	per day	250,000
Hall/venue rental, medium size, National level	per day	150,000
Hall/venue rental, small size, National level	per day	100,000
Hall/venue rental, large size, Regional level	per day	100,000
Refreshments (coffee, snacks)- National level	Per person per day	2,500
Refreshments (coffee, snacks)- Region/District/HF	Per person per day	1,000

Lunch - National level	Per person per day	5,000
Lunch - Regional level	Per person per day	2,500
Lunch - District/HF level	Per person per day	1,500
Cocktail	Per person per day	12,500
Fuel- reimbursement at National and regional level	Reimbursement per KM	105
Fuel- reimbursement at district level to health facility	Reimbursement per KM	40
Vehicle hire, medium	Per vehicle per day	75,000
Vehicle hire, large size (truck)	Per vehicle per day	75,000
Fuel	Per liter	615
Cold chain and equipment [3,4]	**Capacity cm3**	
Cold room, walk-in type,10 m³	10,000,000	15,100
Cold room, walk-in type,30 m³	30,000,000	22,500
Cold room, walk-in type,40 m³	40,000,000	27,000
Mains Ref SureChill GVR50AC E003/046	46,500	1,642
Mains Ref SureChill GVR100AC-M1 E003/047	99,000	2,355
Cold box, RCW 12/CF,PQS E004/004	7,000	614
Mains Ref. Vestfrost VLS400A AC E003/065	145,000	1,322
Mains Ref Aucma MetafridgeCFD50 E003/079	50,000	1,400
Mains Ref Dulas VC225ILR E003/072	184,000	3,318
Mains Ref. Vestfrost VLS400A AC E003/065	145,000	1,322
Mains Ref Aucma MetafridgeCFD50 E003/079	50,000	1,400
Mains Ref SurChl GVR99Lite AC E003/082	98,500	1,200
Mains Ref SureChill GVR225AC-M1 E003/083	225,000	1,910
TCW 2000 AC	60,000	3,034
TCW 3000 AC	150,000	4,346
Cold Box, BlowKing CB/20-CF	20,000	200
TCW15SDD	16,000	4,584
VLS200A	60,000	818
VLS154	170,000	3,140
TCW4000AC	240,000	3,896
VLS400A	145,000	1,118

Sources:

[1] MOH/EPI

[2] Previous new vaccine introduction activity reports from EPI

[3] UNICEF Supply Catalogue (last checked Jan 2018)

[4] Assumptions informed by Kenya Malaria vaccine implementation program costs

Appendix Table 3.a: List of districts and facilities surveyed in Mali

	Name of the Institution/facility
National level	National Center of Immunization (NIC)
	National Malaria Control Program (NMP)
	Technical Advisory Group for Vaccine and Immunization (NITAG or GTCV)
	National Center for Information, Education of the State (CNIECS)
Regional level<br>Health district level	Regional Health Directorate (DRS) of Koulikoro<br>District Health Office, Bougouni
	District Health Office, Diema
Health facility level	Bougouni-Nord
	Bougouni-Sud
	Bougouni-Ouest
	Kaumantou
	Diema-Central
	Diancounte-Camara
	Tinkaré

Appendix Table 3.b: List of districts and facilities surveyed in Burkina Faso

	Name of the Institution/facility
National level	Direction de la Prevention par la Vaccination (DPV)
Regional level<br>Health district level	Region Centre Ouest,<br>Nanoro health district
Health facility level	Secteur 23 Tanghin
	Secteur 27 Wayalghin
	Roumtenga
	Sakoula
	Pella
	Somassi
	Siglè
	Nanoro

Appendix Table 4: Unit cost calculations

Metric	Definition	Calculations
Cost per dose administered	Total cost of the program (both introduction and recurrent), inclusive of commodity costs, divided by the total expected number of vaccinations over a given period of time.	$= \frac{\sum_{i-1}^{n} cost\ of\ activity_i}{Number\ of\ vaccine\ doses\ administered}$<br><br>Where, i = activity groups used in costing
Cost of delivery per dose	Total cost of the program (both introduction and recurrent), less the commodity related cost, divided by the total expected number of vaccinations over a given period of time.	$= \frac{\sum_{i-1}^{n} cost\ of\ activity_i\ - cost\ of\ immunization\ commodity}{Number\ of\ vaccine\ doses\ administered}$<br><br>Where, i = activity groups used in costing<br>Immunization commodities include vaccines, injection supplies, procurement add on charges on these commodities
Cost of delivery per first 3 doses completion	Total cost of the program (both introduction and recurrent), less the commodity related cost, divided by the total expected number of children who complete the first three doses of over a given period of time.	$= \frac{\sum_{i-1}^{n} cost\ of\ activity_i}{Number\ of\ children\ who\ recieve\ atleast\ the\ first\ 3\ doses\ of\ vaccine}$<br><br>Where, i = activity groups used in costing<br>Pri
Cost of delivery per first 3 doses completion	Total cost of the program (both introduction and recurrent), less the commodity related cost, divided by the total expected number of children who complete the first three doses of over a given period of time.	$= \frac{\sum_{i-1}^{n} cost\ of\ activity_i\ - cost\ of\ immunization\ commodity}{Number\ of\ children\ who\ recieve\ atleast\ the\ first\ 3\ doses\ of\ vaccine}$<br><br>Where, i = activity groups used in costing<br>Immunization commodities include vaccines, injection supplies, procurement add on charges on these commodities

Diawara H, *et al. BMJ Global Health* 2023; 8:e011316. doi: 10.1136/bmjgh-2022-011316

Appendix Table 5.a: RTS,S introduction and delivery cost drivers in Mali, scenario 1: Mass campaign

Cost categories	Financial cost			Economic cost		
	USD	%	% w/o commodities	USD	%	% w/o commodities
Vaccines and Injectable Supplies	14,403,323	26.3%	NA	132,402,606	75.6%	NA
Service Delivery	14,384,942	26.3%	35.7%	15,226,456	8.7%	35.6%
Planning and coordination	1,554,067	2.8%	3.9%	1,795,925	1.0%	4.2%
Training	5,209,902	9.5%	12.9%	5,540,074	3.2%	13.0%
Information, Education and Communication	1,076,902	2.0%	2.7%	1,135,973	0.6%	2.7%
Social Mobilization	1,875,034	3.4%	4.7%	1,881,866	1.1%	4.4%
Monitoring and Evaluation	500,012	0.9%	1.2%	500,012	0.3%	1.2%
Supervision	5,447,908	10.0%	13.5%	6,406,462	3.7%	15.0%
Distribution	1,136,620	2.1%	2.8%	1,159,810	0.7%	2.7%
Cold Chain Equipment	3,333,958	6.1%	8.3%	3,333,958	1.9%	7.8%
Other Logistical Equipment	5,743,620	10.5%	14.3%	5,743,620	3.3%	13.4%
**Total cost (full cost, initial investment + recurrent)**	**54,666,288**	**100%**		**175,126,762**	**100%**	
**Total cost (full cost, initial investment + recurrent) without vaccines and injection supplies**	**40,262,966**		**100.0%**	**42,724,156**		**100.0%**

Note: Cost drivers in Mali derived under baseline input assumptions, for mass campaign delivery modality.

Appendix Table 5.b: RTS,S introduction and delivery cost drivers in Mali, scenario 2: Routine EPI

Cost categories	Financial cost			Economic cost		
	USD	%	% w/o commodities	USD	%	% w/o commodities
Vaccines and Injectable Supplies	14,403,323	48.4%	NA	132,402,606	88.9%	NA
Service Delivery	359,339	1.2%	2.3%	1,033,941	0.7%	6.2%
Planning and coordination	327,614	1.1%	2.1%	376,712	0.3%	2.3%
Training	1,962,848	6.6%	12.8%	2,079,762	1.4%	12.6%
Information, Education and Communication	1,007,408	3.4%	6.6%	1,066,478	0.7%	6.4%
Social Mobilization	745,533	2.5%	4.9%	748,283	0.5%	4.5%
Monitoring and Evaluation	20,000	0.1%	0.1%	20,000	0.0%	0.1%
Supervision	1,528,660	5.1%	10.0%	1,794,064	1.2%	10.8%
Distribution	327,157	1.1%	2.1%	347,373	0.2%	2.1%
Cold Chain Equipment	3,333,958	11.2%	21.7%	3,333,958	2.2%	20.2%
Other Logistical Equipment	5,743,620	19.3%	37.4%	5,743,620	3.9%	34.7%
**Total cost (full cost, initial investment + recurrent)**	**29,759,460**	**100%**		**148,946,798**	**100%**	
**Total cost (full cost, initial investment + recurrent) without vaccines and injection supplies**	**15,356,137**		**100.0%**	**16,544,192**		**100.0%**

Diawara H, *et al. BMJ Global Health* 2023; 8:e011316. doi: 10.1136/bmjgh-2022-011316

Note: Cost drivers in Mali derived under baseline input assumptions, for routine EPI delivery modality.

Appendix Table 5.c: RTS,S introduction and delivery cost drivers in Mali, scenario 3: Mixed delivery

Cost categories	Financial cost			Economic cost		
	USD	%	% w/o commodities	USD	%	% w/o commodities
Vaccines and Injectable Supplies	14,403,323	35.7%	NA	132,402,606	82.7%	NA
Service Delivery	3,164,460	7.9%	12.2%	3,872,444	2.4%	14.0%
Planning and coordination	1,554,375	3.9%	6.0%	1,796,268	1.1%	6.5%
Training	5,209,902	12.9%	20.1%	5,540,074	3.5%	20.0%
Information, Education and Communication	1,224,578	3.0%	4.7%	1,283,648	0.8%	4.6%
Social Mobilization	1,875,034	4.7%	7.2%	1,881,866	1.2%	6.8%
Monitoring and Evaluation	500,012	1.2%	1.9%	500,012	0.3%	1.8%
Supervision	2,618,242	6.5%	10.1%	3,075,357	1.9%	11.1%
Distribution	674,945	1.7%	2.6%	701,688	0.4%	2.5%
Cold Chain Equipment	3,333,958	8.3%	12.9%	3,333,958	2.1%	12.0%
Other Logistical Equipment	5,743,620	14.3%	22.2%	5,743,620	3.6%	20.7%
**Total cost (full cost, initial investment + recurrent)**	**40,302,449**	**100%**		**160,131,541**	**100%**	
**Total cost (full cost, initial investment + recurrent) without vaccines and injection supplies**	**25,899,126**		**100.0%**	**27,728,935**		**100.0%**

Note: Cost drivers in Mali derived under baseline input assumptions, for mixed delivery modality.

Appendix Table 6.a: RTS,S introduction and delivery cost drivers in Burkina Faso, scenario 1: Mass campaign

Cost categories	Financial cost			Economic cost		
	USD	%	% w/o commodities	USD	%	% w/o commodities
Vaccines and Injectable Supplies	13,647,445	41.8%	NA	125,454,195	84.8%	NA
Service Delivery	10,404,650	31.9%	54.8%	12,796,524	8.7%	57.0%
Planning and coordination	995,501	3.0%	5.2%	1,426,266	1.0%	6.4%
Training	434,792	1.3%	2.3%	547,837	0.4%	2.4%
Initial Sensitization	84,044	0.3%	0.4%	139,710	0.1%	0.6%
Communication	186,940	0.6%	1.0%	186,940	0.1%	0.8%
Social Mobilization	23,976	0.1%	0.1%	32,476	0.0%	0.1%
Monitoring and Evaluation	135,274	0.4%	0.7%	167,098	0.1%	0.7%
Supervision	1,094,491	3.4%	5.8%	1,363,940	0.9%	6.1%
Distribution	549,530	1.7%	2.9%	699,601	0.5%	3.1%
Cold Chain Equipment	5,091,141	15.6%	26.8%	5,091,141	3.4%	22.7%
**Total cost (full cost, initial investment + recurrent)**	**32,647,782**	**100.0%**		**147,905,729**	**100.0%**	

**Total cost (full cost, initial investment + recurrent) without vaccines and injection supplies**	**19,000,337**		**100.0%**	**22,451,534**		**100.0%**

Note: Cost drivers in Burkina Faso derived under baseline input assumptions, for mass campaign delivery modality.

Appendix Table 6.b: RTS,S introduction and delivery cost drivers in Burkina Faso, scenario 2: Routine EPI

Cost categories	Financial cost			Economic cost		
	USD	%	% w/o commodities	USD	%	% w/o commodities
Vaccines and Injectable Supplies	13,647,445	64.5%	NA	125,454,195	93.2%	NA
Service Delivery	572,615	2.7%	7.6%	1,415,750	1.1%	15.5%
Planning and coordination	399,165	1.9%	5.3%	596,612	0.4%	6.5%
Training	222,374	1.1%	3.0%	316,108	0.2%	3.5%
Initial Sensitization	84,044	0.4%	1.1%	139,710	0.1%	1.5%
Communication	58,236	0.3%	0.8%	58,236	0.0%	0.6%
Social Mobilization	23,976	0.1%	0.3%	32,476	0.0%	0.4%
Monitoring and Evaluation	135,274	0.6%	1.8%	167,098	0.1%	1.8%
Supervision	733,968	3.5%	9.8%	1,117,069	0.8%	12.2%
Distribution	183,722	0.9%	2.4%	218,312	0.2%	2.4%
Cold Chain Equipment	5,091,141	24.1%	67.8%	5,091,141	3.8%	55.6%
**Total cost (full cost, initial investment + recurrent)**	**21,151,958**	**100.0%**		**134,606,708**	**100.0%**	
**Total cost (full cost, initial investment + recurrent) without vaccines and injection supplies**	**7,504,513**		**100.0%**	**9,152,513**		**100.0%**

Note: Cost drivers in Burkina Faso derived under baseline input assumptions, for routine EPI delivery modality.

Appendix Table 6.c: RTS,S introduction and delivery cost drivers in Burkina Faso, scenario 3: Mixed delivery

Cost categories	Financial cost			Economic cost		
	USD	%	% w/o commodities	USD	%	% w/o commodities
Vaccines and Injectable Supplies	13,647,445	55.3%	NA	125,454,195	90.3%	NA
Service Delivery	2,539,022	10.3%	23.1%	3,691,905	2.7%	27.4%
Planning and coordination	1,159,656	4.7%	10.5%	1,684,445	1.2%	12.5%
Training	434,792	1.8%	3.9%	547,837	0.4%	4.1%
Initial Sensitization	168,087	0.7%	1.5%	279,420	0.2%	2.1%
Communication	186,940	0.8%	1.7%	186,940	0.1%	1.4%
Social Mobilization	23,976	0.1%	0.2%	32,476	0.0%	0.2%

Monitoring and Evaluation	135,274	0.5%	1.2%	167,098	0.1%	1.2%
Supervision	1,025,832	4.2%	9.3%	1,480,787	1.1%	11.0%
Distribution	247,704	1.0%	2.2%	288,248	0.2%	2.1%
Cold Chain Equipment	5,091,141	20.6%	46.2%	5,091,141	3.7%	37.9%
**Total cost (full cost, initial investment + recurrent)**	**24,659,868**	**100.0%**		**138,904,492**	**100.0%**	
**Total cost (full cost, initial investment + recurrent) without vaccines and injection supplies**	**11,012,423**		**100.0%**	**13,450,297**		**100.0%**

Note: Cost drivers in Burkina Faso derived under baseline input assumptions, for mixed delivery modality.

Appendix Table 7: Unit cost estimates (in USD) at various vaccine price assumptions

**Metric**	**Vaccine price per dose**	**Sn1: Mass campaign**		**Sn2: Routine EPI**		**Sn3: Mixed delivery**	
		Financial	Economic	Financial	Economic	Financial	Economic
**Mali**							
Cost per dose administered	$2	$2.34	$4.80	$1.10	$3.50	$1.62	$4.05
Cost per dose administered	$5	$2.71	$8.86	$1.47	$7.38	$2.00	$7.93
Cost per dose administered	$10	$3.33	$15.14	$2.09	$13.84	$2.62	$14.40
Cost per first 3 doses completion	$2	$9.98	$21.93	$4.03	$15.74	$7.39	$19.21
Cost per first 3 doses completion	$5	$11.08	$40.41	$5.80	$34.22	$9.16	$37.69
Cost per first 3 doses completion	$10	$14.71	$71.22	$8.75	$65.03	$12.12	$68.51
**Burkina Faso**							
Cost per dose administered	$2	$1.33	$3.85	$0.73	$3.16	$0.92	$3.38
Cost per dose administered	$5	$1.71	$7.73	$1.11	$7.04	$1.29	$7.26
Cost per dose administered	$10	$2.33	$14.19	$1.73	$13.50	$1.91	$13.72
Cost per first 3 doses completion	$2	$5.60	$17.72	$2.73	$14.41	$3.61	$15.48
Cost per first 3 doses completion	$5	$7.37	$36.23	$4.51	$32.91	$5.38	$33.98
Cost per first 3 doses completion	$10	$10.33	$67.07	$7.46	$63.76	$8.34	$64.82

Appendix Figure 1.a: Financial unit cost estimates across different scenarios

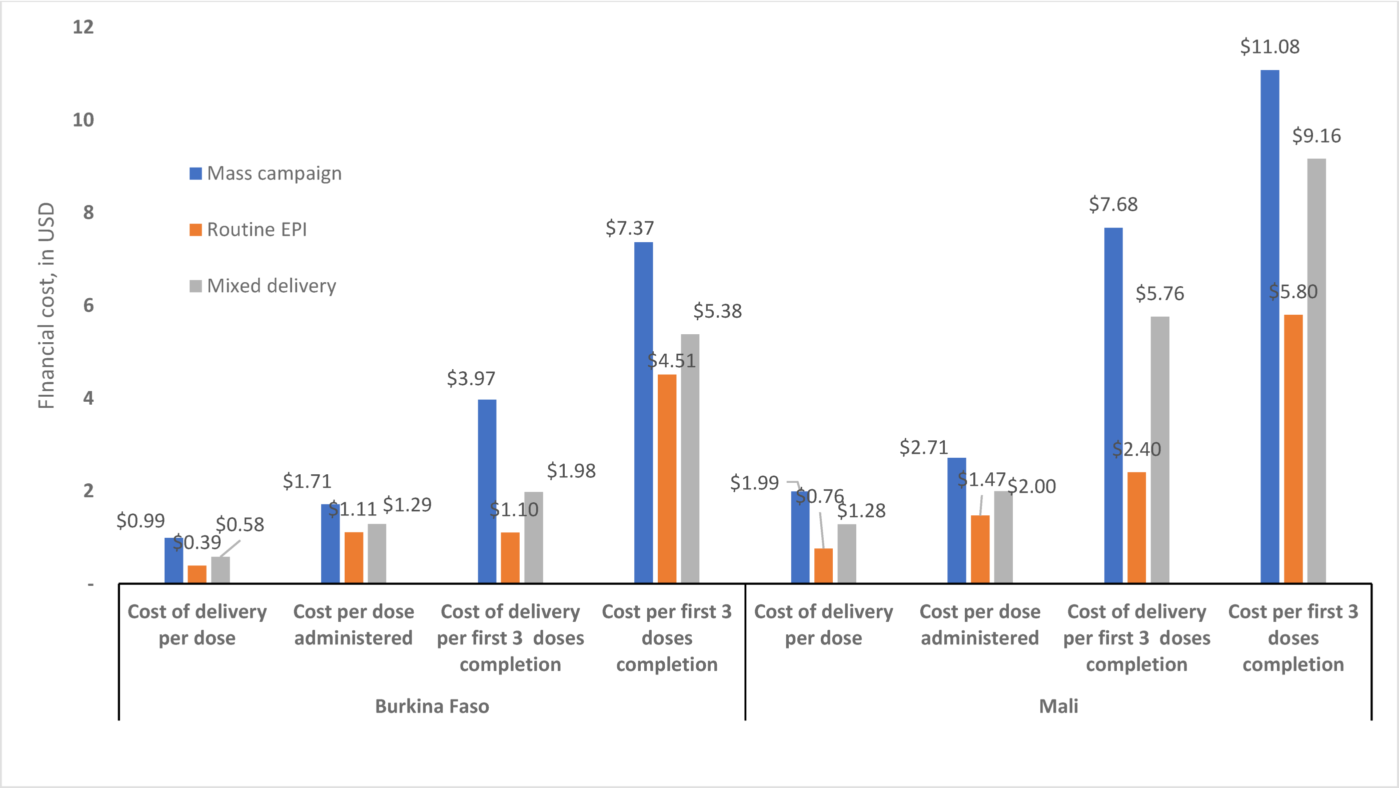

Appendix Figure 1.b: Economic unit cost estimates across different scenarios

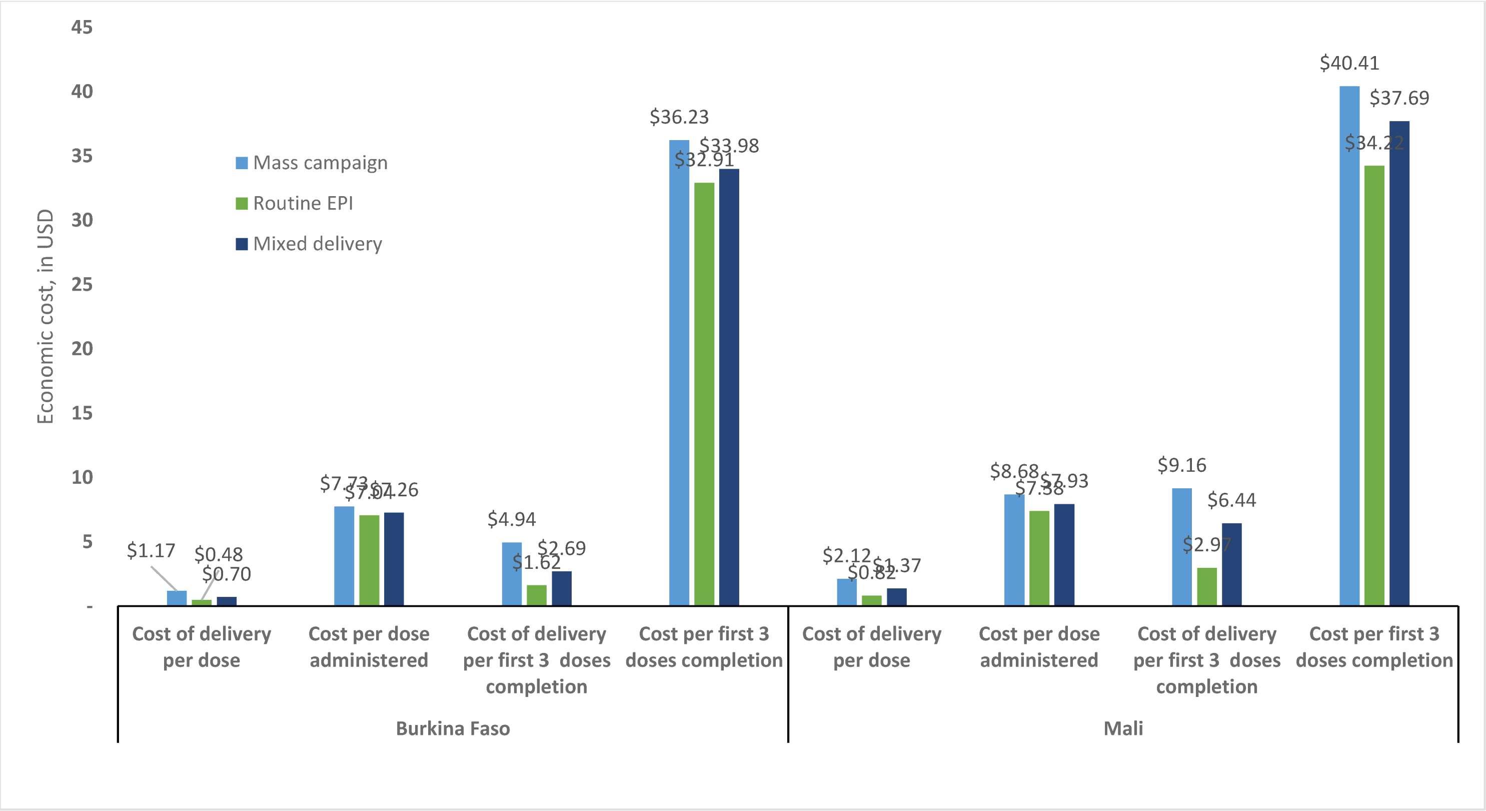

Diawara H, et al. BMJ Global Health 2023; 8:e011316. doi: 10.1136/bmjgh-2022-011316

Appendix Figure 2.a: Cost drivers across different scenarios of vaccine delivery, Burkina Faso

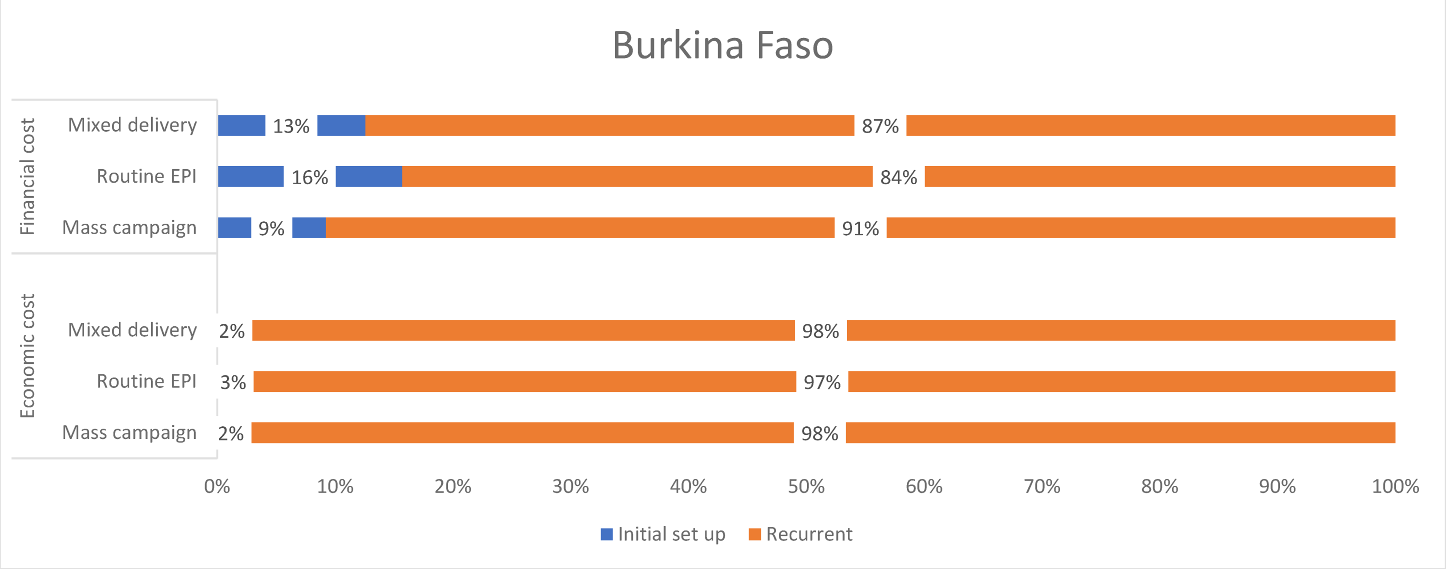

Appendix Figure 2.b: Cost drivers across different scenarios of vaccine delivery, Mali

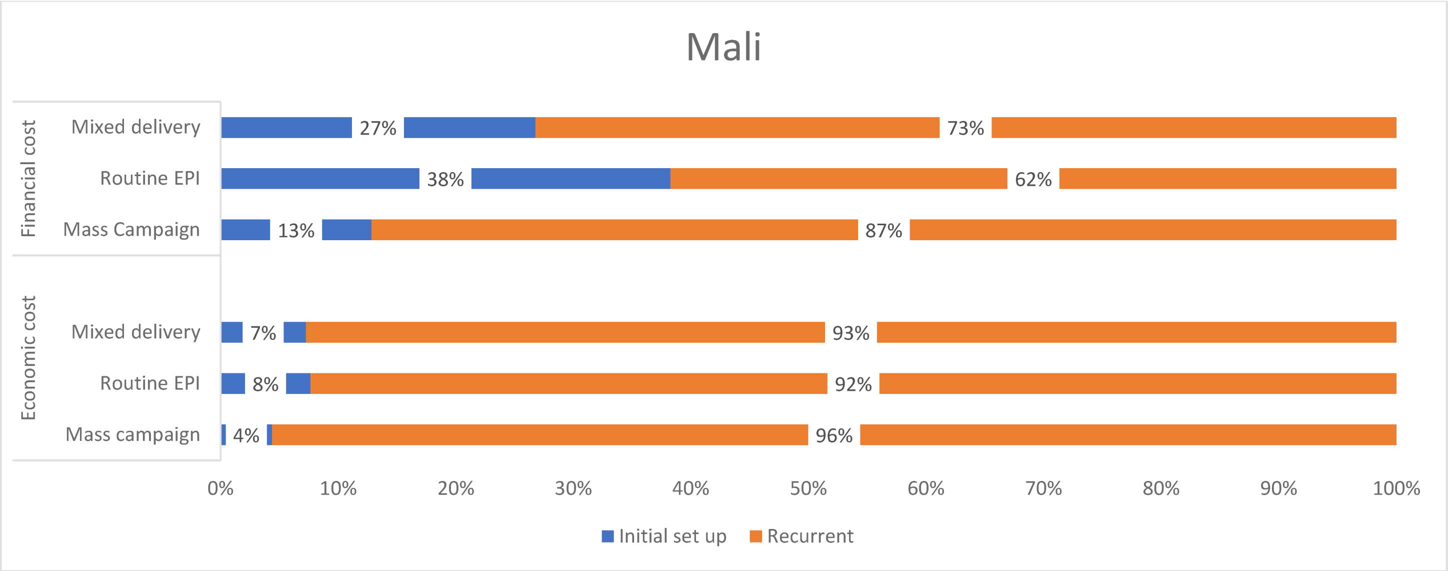

Diawara H, et al. BMJ Global Health 2023; 8:e011316. doi: 10.1136/bmjgh-2022-011316

BMJ Publishing Group Limited (BMJ) disclaims all liability and responsibility arising from any reliance placed on this supplemental material which has been supplied by the author(s)

Diawara H, *et al. BMJ Global Health* 2023; 8:e011316. doi: 10.1136/bmjgh-2022-011316
