# Supplementary material for: Cost of introducing and delivering malaria vaccine (RTS, S/AS01E) in areas of seasonal malaria transmission, Mali and Burkina Faso
Source: BMJ Glob Health. 2023 Apr 17;8(4):e011316. doi: 10.1136/bmjgh-2022-011316 (PMC10111920; doi:10.1136/bmjgh-2022-011316)
Supplement: Supplementary data [file bmjgh-2022-011316supp002.pdf]

Appendix S1 – Reflexivity Statement

1. How does this study address local research and policy priorities?
Malaria parasite is highly seasonal across the African Sahel sub-region including Burkina Faso and Mali. This study aimed to understand the cost of malaria vaccine delivery for different delivery modalities, Burkina Faso and Mali. As these countries consider implementation of the RTS,S vaccine into their immunization programs, the economic evidence generated in this study can be used by countries to help guide their decisions around RST,S vaccine introduction and choice of delivery modality, by evaluating the economic implications of each strategy.

2. How were local researchers involved in study design?
This study was done by an international partnership of researchers from high-income and low-income countries. The conceptualization of research was done from partners from high-income countries. The researchers from low-income countries led the data collection, engagement within country program managers and leaders to validate the data. All authors contributed to the interpretation of the data and review of the manuscript. The roles of each co-author are clearly stated in the "contributions of each person" section.

3. How has funding been used to support the local research team?
The funding for this study was supported by PATH with institutional agreement with the in-country researchers in Burkina Faso and Mali.

4. How are research staff who conducted data collection acknowledged?
Researchers leading the data collection are co-authors in this study. Research staff/data collectors are acknowledged in the acknowledgment section.

5. Do all members of the research partnership have access to study data?
All members of the partnership have access to data.

6. How was data used to develop analytical skills within the partnership?
The partners are lead health economists in their respective countries and contributed to the analysis and interpretation of the results.

7. How have research partners collaborated in interpreting study data?
The research partners collaborated in interpretation of the data via joint conference calls throughout the analysis period. Further, they led the validation meeting within each country with the in-country program managers and decision makers to interpret the data.

8. How were research partners supported to develop writing skills?
This was not specifically addressed during this study.

9. How will research products be shared to address local needs?
Validation and dissemination workshop in each country was done by in-country researchers. The workshop included in-country decision makers, program managers, malaria academic institutions in country. This paper will be published as open access.

10. How is the leadership, contribution and ownership of this work by LMIC researchers recognised within the authorship?

The study implementation was led by researchers in low-income countries. Majority of authors including the joint first co-authors in this manuscript (HD and FB) are from low-income countries reflecting the critical leadership, contribution, and ownership of this work.

11. **How have early career researchers across the partnership been included within the authorship team?**

We have included early career researchers (FK, RG, SY, and ST) within the authorship team.

12. How has gender balance been addressed within the authorship?

Seven authors are female (HD, FB, AL, CL, FK, RG, and RB) and six authors male (AD, JO, SY, ST, WM, and CP).

13. How has the project contributed to training of LMIC researchers?

The authorship team is primarily composed of senior researchers. All the authors based in low-income countries mostly senior researchers. Research funding leveraged as part of this project supported employment of a few junior researcher in Burkina Faso and Mali.

14. How has the project contributed to improvements in local infrastructure?

This project has not directly contributed to improvements in local infrastructure.

15. What safeguarding procedures were used to protect local study participants and researchers?

Study participants in this study included the health workers and their participation in the study pose minimal risk to interviewees. Further, all participants provided informed consent, and we have anonymized all participants.
